# Operative Treatment of Adolescent Diaphyseal Clavicle Fracture: Elastic Stable Intramedullary Nail Versus Plate Fixation

**DOI:** 10.3390/medicina61081494

**Published:** 2025-08-21

**Authors:** Kunhyung Bae, Soorack Ryu, Sung Hoon Choi, Hyunjae Kwon, Yoon Hae Kwak

**Affiliations:** 1 Department of Orthopaedic Surgery, Hanyang University Hospital, Hanyang University College of Medicine, 222 Wangsimni-ro, Seongdong-gu, Seoul 04763, Republic of Korea; 2 Biostatistical Consulting and Research Laboratory, Medical Research Collaborating Center, Hanyang University, Seoul 04763, Republic of Korea; 3 Department of Orthopaedic Surgery, Asan Medical Center Children’s Hospital, University of Ulsan College of Medicine, 88, Olympic-ro, 43-gil, Songpa-gu, Seoul 05505, Republic of Korea; d250659@amc.seoul.kr

**Keywords:** adolescent, diaphyseal clavicle fracture, elastic stable intramedullary nail, plate fixation

## Abstract

*Background and Objectives*: Adolescent diaphyseal clavicle fracture surgery has increased in recent years. However, the optimal operative method remains debated, particularly between elastic stable intramedullary nailing (ESIN) and plate fixation. This study compared postoperative outcomes and complication rates between ESIN and plate fixation for treating diaphyseal clavicle fractures in adolescent patients. *Materials and Methods*: We conducted a retrospective review of 35 adolescents who underwent surgery for diaphyseal clavicle fractures between 2010 and 2024. Patients were assigned to either the ESIN group (*n* = 18) or the plate fixation group (*n* = 17). Postoperative outcomes assessed included the Quick Disabilities of the Arm, Shoulder, and Hand (QuickDASH) score, intraoperative time, time to complete fracture union, and clavicle shortening at 1 year postoperatively. Postoperative complications were also evaluated. *Results*: Fracture union occurred significantly faster with ESIN than with plate fixation, specifically 3 weeks earlier (11.0 weeks vs. 14.0 weeks; *p* < 0.001). No significant differences were observed between the groups in QuickDASH scores, intraoperative time, or clavicle shortening at 1 year. The overall postoperative complication rate was 25.7% (9/35), with no statistically significant difference between the ESIN (27.8%) and plate fixation groups (23.5%) (*p* = 0.774). Refractures occurred exclusively in the plate fixation group (*n* = 2), while one patient in the ESIN group required early nail removal due to insertion site irritation. *Conclusions*: ESIN provided functional and radiographic outcomes comparable to plate fixation in adolescents with diaphyseal clavicle fractures, with a 3-week shorter time to union and a less-invasive surgical approach.

## 1. Introduction

Clavicle fractures are among the most common traumatic injuries in the adolescent population, with diaphyseal lesions accounting for approximately 90% of cases and up to 45% of all shoulder-girdle fractures [1,2]. The incidence peaks in male adolescents around the age of 14 years, with most injuries resulting from contact or overhead sports, falls, or direct blows to the shoulder [3]. The thick periosteum and considerable remodeling capacity of the adolescent clavicle support high union rates with simple sling immobilization, making it the recommended first-line treatment [4].

Although conservative treatment has been considered for most adolescent diaphyseal clavicle fractures, severely displaced or shortened fractures may be related to functional impairment, leading to shoulder weakness, especially in active adolescents with higher functional demands. Therefore, the increasing participation of young individuals in competitive sports and a greater emphasis on rapid return to activity have contributed to the growing preference for surgical treatment in this age group in recent years. Recent epidemiological studies have also shown an increase in surgical fixation rates for adolescent clavicle fractures, reflecting changes in both patient expectations and surgeon preference [5].

The absolute indications for surgical treatment include open fractures, threatened or compromised skin integrity, neurovascular injury, and floating shoulder trauma. However, the role of operative fixation for significantly displaced or shortened fractures in adolescents remains controversial [6]. While recent studies suggest that surgical treatment may promote bone healing, enhance functional recovery, and reduce complication rates [7,8], evidence supporting these benefits in adolescents is limited. Furthermore, the limitations of existing research, such as retrospective study designs, small sample sizes, heterogeneous surgical techniques, and variable follow-up durations, restrict the generalizability of the findings.

Current surgical options include plate fixation, which provides stable anatomical reduction and allows for early mobilization, but requires a longer incision and is associated with implant prominence, soft-tissue irritation, or potential supraclavicular nerve injury. In contrast, elastic stable intramedullary nailing (ESIN) is minimally invasive, offers easier hardware removal, and is generally associated with better cosmetic results. However, ESIN may provide less rotational stability and carries a risk of implant migration or skin irritation at the entry point. Most existing comparative studies have focused on adult populations, whereas comparative studies evaluating surgical outcomes specifically in adolescents remain limited [9]. Because adolescents differ from adults in terms of bone biology, remodeling potential, and postoperative activity levels, it is uncertain whether the findings from adult studies can be directly applied to this age group.

Therefore, this study aimed to compare the radiological and functional outcomes between ESIN and plate fixation in adolescent diaphyseal clavicle fractures. Additionally, postoperative complication rates associated with each surgical method were analyzed to identify the safer operative approach for this age group.

## 2. Materials and Methods

We retrospectively reviewed the medical records of patients with adolescent diaphyseal clavicle fractures who attended our pediatric orthopedic clinic at a tertiary hospital between 2010 and 2024. A total of 985 patients were identified during this period. The inclusion criteria were as follows: (i) patients aged 12–18 years diagnosed with diaphyseal clavicle fractures; (ii) patients who underwent surgical treatment in accordance with our institutional indications; and (iii) patients who underwent implant removal and completed at least 1 year of postoperative follow-up. The exclusion criteria were as follows: (i) congenital or neuromuscular conditions affecting the musculoskeletal system; (ii) prior fracture of the same clavicle; (iii) bilateral clavicle fractures; and (iv) delayed surgery for malunion or nonunion following failed conservative treatment. Ultimately, 35 patients met all criteria and were included in the final analysis. A post hoc power analysis was performed using the observed difference between the ESIN and plate fixation groups (Cohen’s d = 1.49). With a total sample size of 35 and a significance level of 0.05, the calculated power was 0.99. The study received approval from the Institutional Review Board (IRB) of Asan Medical Center (IRB no. 2025-0335).

### 2.1. Operative Indications, Surgical Techniques, and Postoperative Management

Absolute indications for surgery included open fractures or threatened skin integrity (e.g., skin tenting or compromise). Relative indications included clavicle shortening >2 cm with segmental fractures or comminuted fractures in patients aged ≥12 years, where surgical or conservative management could be selected based on clinical judgment. Seventeen patients underwent surgery for absolute indications and 18 for relative indications.

Two surgical methods were employed for the treatment of the adolescent diaphyseal clavicle fracture. ESIN was performed using 2.0–3.0 mm Titanium Elastic Nails (DePuy Synthes, West Chester, PA, USA). A minimal (approximately 2 cm) incision was used for open reduction at the fracture site. A secondary proximal clavicle incision (1 cm medial) was made, and an awl was used to create the medullary canal entry. The nail was inserted and passed the fracture site under direct vision to avoid damage to the surrounding structures. Plate fixation involved the use of 2.7 mm or 3.5 mm LCP Superior Anterior Clavicle Plates (DePuy Synthes, West Chester, PA, USA), selected based on clavicle size and fracture type. A 6–8 cm longitudinal incision along the superior clavicle enabled direct reduction and fixation with a minimum of three screws on each side, ensuring engagement of at least six cortices. Surgical method selection was based on the surgeon’s experience and judgement (YHK).

Postoperative management started with arm sling immobilization, and early pendulum exercises were encouraged as tolerated to prevent joint stiffness. At 2 weeks postoperatively, the patients were instructed to remove the sling indoors and begin active shoulder exercises within a pain-free range. Heavy lifting and elevation of the arm above 90° in forward flexion or abduction were restricted, and the use of the sling was maintained outdoors until 4 weeks postoperatively. Radiographic evaluation was conducted preoperatively and at 4 weeks to assess callus formation. Active shoulder motion and return to daily activities were encouraged. Follow-up radiographs were obtained every 2 weeks to monitor healing and guide the return to sports upon confirmed union. Implants were routinely removed approximately 1 year postoperatively after confirmed fracture union, in line with our institutional protocol. This approach reflects the preference of many patients and guardians in South Korea to remove hardware and aims to reduce potential complications during adolescence. All patients agreed to this protocol, resulting in a 100% implant removal rate, unless earlier removal was required due to symptomatic implant irritation.

### 2.2. Investigated Variables

**Figure 1:**
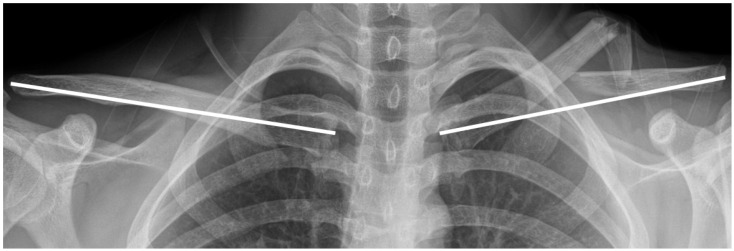
Clavicle length was measured from the most lateral to the most medial point of the anteroposterior radiograph. The length of the fractured clavicle was compared with that of the uninjured contralateral side for the calculation of clavicle shortening (both white lines).

Preoperative variables included patient age, sex, body mass index (BMI), fracture laterality, and injury severity. Injuries were categorized as high- or low-energy; high-energy was defined as resulting from road-traffic accidents or falls from heights > 3 m [10]. Radiological assessments included the identification of fracture type using the AO/OTA classification system (15-A for simple fracture, 15-B for wedge fracture, and 15-C for segmentary fracture) and clavicle shortening. Shortening was measured on clavicle anteroposterior (AP) radiographs by comparing the injured side with the contralateral, uninjured clavicle (Figure 1) [11]. Clavicle shortening measurements were obtained preoperatively and at 1-year follow-up. Inter- and intra-observer reliability, evaluated using the intraclass correlation coefficient (ICC), indicates excellent consistency if it is above 0.90.

Postoperative outcomes included (i) intraoperative time (from skin incision to closure); (ii) time to complete fracture union, defined by cortical bridging on both AP and cephalic-tilt views between the proximal and distal fragments; and (iii) residual clavicle shortening at 1-year follow-up. Functional outcomes were assessed using the Quick Disabilities of the Arm, Shoulder, and Hand (QuickDASH) questionnaire at the 1-year postoperative visit [12,13]. Postoperative complications assessed included implant irritation, refracture following implant removal, chest wall numbness, superficial wound infection, and implant bending or breakage by operative methods.

### 2.3. Statistical Analysis

Continuous variables were expressed as medians with interquartile ranges (IQR). Comparisons between treatment groups were performed using the Mann–Whitney U test. Categorical variables were presented as frequencies with percentages and compared using the Chi-square or Fisher’s exact test, as appropriate. Statistical significance was defined as a *p*-value < 0.05. All statistical analyses were conducted using IBM SPSS Statistics, version 21.0 (IBM Co., Armonk, NY, USA).

## 3. Results

A total of 35 patients were included, with a median age of 14.9 (IQR: 14.3, 15.8) years. Of those, 32 (91.4%) were male, and the median BMI was 22.4 (IQR: 19.1, 24.1) kg/m^2^. A total of 18 fractures were treated with ESIN, and 17 fractures were treated with plate fixation. There was one case of a pinpoint open fracture (Gustilo–Anderson type I), caused by a segmental fragment perforating the skin. This patient was managed with immediate debridement and fixation using ESIN with no delay to treatment, which did not result in any postoperative complications.

There were no statistically significant differences in the baseline demographic characteristics and preoperative radiographic parameters between the two groups (Table 1).

Regarding postoperative outcomes, operative time was similar between the ESIN and plate fixation groups (62.5 min [IQR: 55.0, 80.0] vs. 65.0 min [60.0, 80.0], *p* = 0.561). However, the time to complete fracture union was significantly shorter in the ESIN group by approximately 3.0 weeks (11.0 [IQR: 10.0, 14.0] vs. 14.0 weeks [IQR: 12.0, 16.0], *p* < 0.001). Clavicular shortening at 1 year postoperatively (2.0 mm [IQR: 0.0, 5.0] vs. 0.0 mm [IQR: −3.0, 4.0], *p* = 0.411) and QuickDASH scores (0.0 [IQR: 0.0, 4,5] vs. 4.5 [IQR: 0.0, 6.8], *p* = 0.339) did not differ significantly between the two groups (Table 2).

The overall postoperative complication rate was 25.7% (9/35), with 23.5% (4/17) in the plate fixation group and 27.8% (5/18) in the ESIN group (*p* = 0.774). There was no statistically significant difference in each postoperative complication between these two groups (Table 3).

**Figure 2:**
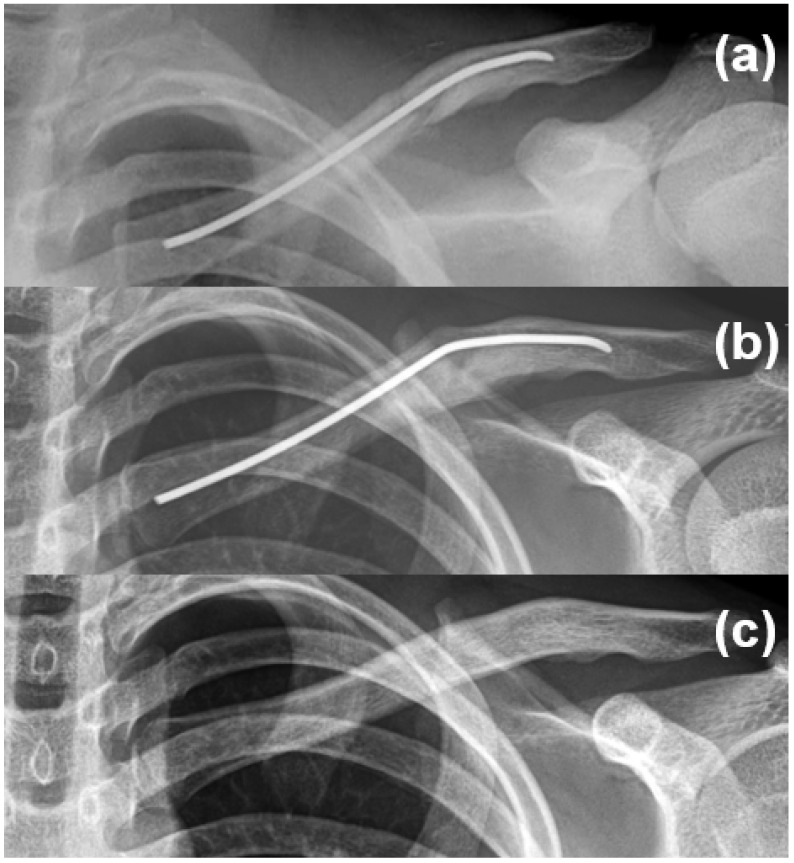
A 14-year-old male patient underwent intramedullary nail fixation for a diaphyseal clavicle fracture (**a**). Implant bending was observed at 1 month postoperatively, without skin irritation, functional limitation, or implant breakage (**b**). The implant was electively removed at 1 year without complications (**c**).

ESIN-related postoperative complications included implant irritation (*n* = 3), superficial wound infection (*n* = 1), and implant bending (*n* = 1) (Figure 2).

**Figure 3:**
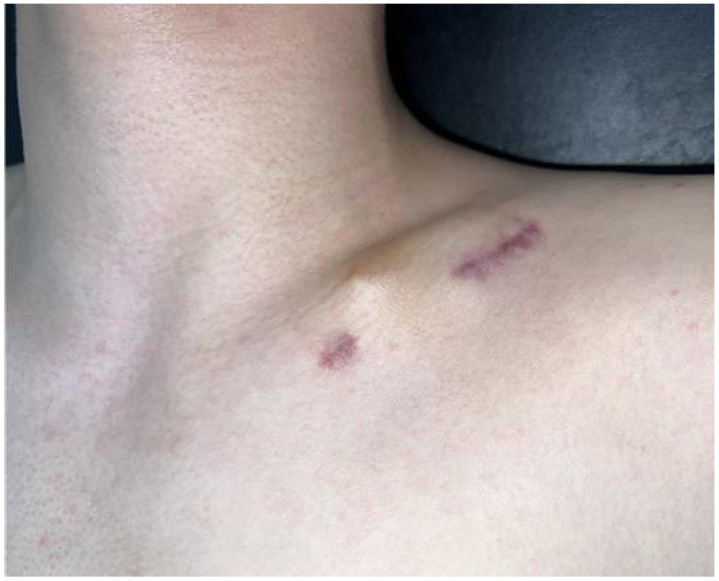
A 15-year-old male patient underwent intramedullary nail fixation for a diaphyseal clavicle fracture. Skin irritation developed at the nail insertion site at 1 month postoperatively, necessitating early implant removal.

One patient treated with ESIN required early implant removal only after 1 month postoperatively due to insertion site irritation (Figure 3).

**Figure 4:**
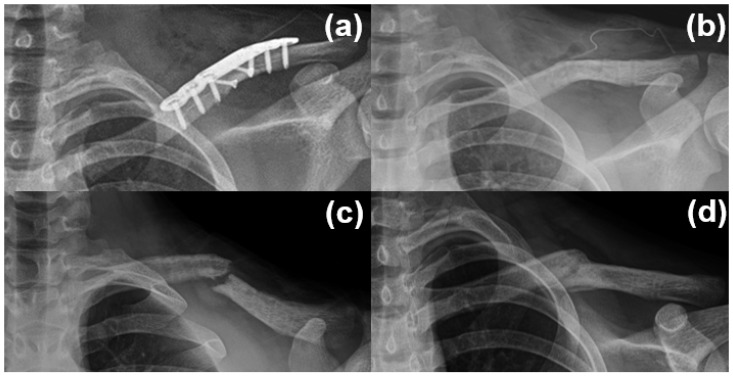
A 14-year-old male patient with a diaphyseal clavicle fracture underwent plate fixation (**a**), followed by plate removal 1 year postoperatively after confirmation of bone union (**b**). A refracture occurred 1 month later due to minor trauma (**c**). Conservative treatment using a figure-of-eight brace led to successful bone union within 3 months (**d**).

In the plate fixation group, postoperative complications included implant irritation (*n* = 1), chest wall numbness (*n* = 1), and refracture (*n* = 2) following implant removal. Both refractures were managed conservatively, and union was achieved without further surgical intervention (Figure 4).

## 4. Discussion

This retrospective study found that plate fixation and ESIN showed comparable functional outcomes and clavicle length restoration at 1-year follow-up postoperatively. The only significant difference was the shorter time to complete fracture union in the ESIN group. Postoperative complications occurred in approximately one in four patients, with the overall complication rates relatively similar between the two groups. Two cases of refracture were observed exclusively in the plate fixation group. However, ESIN-related complications led to early implant removal in one patient due to irritation of the skin at the nail insertion site, and there was one case of implant bending. These findings suggest that ESIN can provide outcomes as favorable as plate fixation while offering a less invasive technique and promoting faster bone union.

### 4.1. Adolescent Clavicle Fracture and Its Treatment Methods

The adolescent clavicle possesses a thick periosteum and considerable remodeling capacity, generally favoring a conservative management of fractures and lowering the risk of non-union. However, longitudinal clavicle growth is approximately 80% complete by age ≤ 12 years [14]. As skeletal maturity approaches, the remaining remodeling potential decreases, making residual deformity more likely in older adolescents compared with younger patients. Additionally, as many adolescents participate in high-demand sports, the need for rapid functional recovery has contributed to a steady increase in operative fixation over the past decade [15]. Cole et al. reported this trend to be most prominent among older male adolescents, where concerns regarding activity level and limited remaining remodeling potential often prompt surgeons to favor surgical intervention [5]. Despite this trend, there remains no established consensus on the optimal approach for managing diaphyseal fractures in this population. The American Academy of Orthopedic Surgeons Clinical Practice Guideline for clavicle shaft fracture provides a moderate recommendation for both plate fixation and ESIN, citing comparable long-term outcomes and postoperative complication rates [16].

### 4.2. Postoperative Outcomes in Adolescent Diaphyseal Clavicular Fracture

Our study revealed that there were no significant differences in postoperative outcomes between ESIN and plate fixation, except for the time to complete fracture union. The QuickDASH scores were favorable in both groups, aligning with prior studies that report excellent postoperative shoulder function regardless of surgical method [9,17]. The QuickDASH score was originally developed to evaluate upper-extremity function in adult populations. However, its applicability has been demonstrated in younger populations, particularly in older children and adolescents. In the present study, we encouraged early pendulum exercises without delay to prevent postoperative joint stiffness. Additionally, at 2 weeks postoperatively, patients were instructed to remove the sling while indoors and to begin active shoulder exercises within a pain-free range. This rehabilitation protocol allowed for an optimal balance between protecting the fracture site and promoting early mobilization, resulting in no cases of shoulder stiffness. These factors may have contributed to the favorable QuickDASH scores observed in both groups, regardless of the surgical method used. Additionally, clavicle shortening at 1-year follow-up was <3 mm in both groups. In contrast to earlier reports associating ESIN with greater clavicle shortening in adults [18], we believe the residual growth potential in adolescents mitigates the clinical consequences of minor length discrepancies [19]. Even when shortening occurs, it does not necessarily affect strength, range of motion, or patient satisfaction in skeletally immature individuals [20]. In our cohort, ESIN achieved fracture union approximately 3 weeks earlier than plate fixation. Accelerated bone healing with ESIN in clavicle fractures has previously been attributed to preservation of the periosteal blood supply and minimal soft-tissue disruption, due to its less-invasive approach to the fracture site [21]. This faster bone healing may provide clinical advantages for patients, such as earlier return to sports, reduced analgesic use, and quicker discharge from physical therapy. Nonetheless, the clinical relevance of these biological advantages was not explored in this study. Therefore, future prospective studies incorporating functional and performance-based endpoints are required to determine the practical significance of accelerated unions, particularly in high-demand adolescent populations. Although prior studies described shorter operative durations with ESIN [9,22], we observed no significant difference between the groups. This may reflect our routine application of a mini-open reduction technique with ESIN for direct fracture reduction. However, we consider this approach advantageous, as it enables clear visualization of the fracture, which is important for accurate anatomical alignment and secure nail placement, while reducing radiation exposure and minimizing the risk of injury to adjacent structures during the procedure [23].

### 4.3. Postoperative Complications in Adolescent Diaphyseal Clavicular Fracture

The overall postoperative complication rate was 25%, consistent with the previously reported range of 16–89% [24,25]. The complication rates were comparable between the two surgical groups, with 27.8% in the ESIN group and 23.5% in the plate fixation group, demonstrating no statistically significant difference. This finding aligns with prior research indicating no substantial disparity in complication rates between these two techniques [25]. Implant irritation occurred in three cases in the plate fixation group and in one case in the ESIN group. However, only one ESIN case required early implant removal due to discomfort. Skin irritation is a recognized issue with ESIN, and patient-reported discomfort is frequently documented. In this study, only one patient (1/17; 5.9%) reported irritation severe enough to necessitate early removal—a relatively low rate compared to other studies [26,27,28] and comparable to that reported by Meijden et al. [29]. To minimize implant-related irritation, the nail end should be rotated post-fixation to align closely with the bone’s contour and to be adequately covered by the platysma muscle to reduce prominence. Chest wall numbness is a well-documented complication associated with midshaft clavicular surgery, commonly resulting from supraclavicular nerve injury, particularly in plate fixation. Although this condition is typically self-limiting, caution remains necessary [30]. In our study, only one case of chest wall numbness was reported in the plate fixation group. The use of a smaller incision (approximately 6–8 cm) and limited soft-tissue dissection for plate exposure can reduce the risk of supraclavicular nerve injury [31]. In addition, any transient sensory disturbances may have resolved by the time of our assessment, which was performed 1 year postoperatively. Moreover, adolescents may exhibit faster and more complete recovery from iatrogenic nerve injuries than adults, owing to their superior healing potential. Refracture occurred in two patients following implant removal in the plate fixation group, in line with previous findings indicating that refractures are more frequently associated with plate fixation [32,33]. Biomechanical studies have demonstrated that screw holes left after plate removal may weaken the clavicle’s structural integrity, increasing refracture risk [34,35]. Both cases were managed conservatively, as bone union had been confirmed prior to hardware removal, suggesting sufficient biological healing potential for non-operative management. One case of implant bending occurred in the ESIN group. Given the higher incidence of adolescent clavicle fractures in males and their elevated activity levels, caution is warranted regarding this complication in patients treated with ESIN. To prevent such occurrences, we recommend adequate immobilization during the early postoperative period until sufficient callus formation is evident, along with good patient compliance, rather than premature or overly aggressive rehabilitation. Although pin migration can result in life-threatening complications with some intramedullary devices, no instances of nail migration occurred in our case series, likely due to the stable, three-point fixation achieved by titanium elastic nails within the S-shaped clavicle.

This study had some limitations. First, the sample size was relatively small, including only 35 patients. Nonetheless, we were able to detect statistically significant differences, such as the time to complete fracture union. Moreover, as conservative treatment is generally preferred for adolescent clavicle fractures, assembling a large cohort from a single center is inherently challenging. In addition, the single-center design may limit the generalizability of our findings, as institution-specific surgical techniques, postoperative care protocols, and rehabilitation protocols could have influenced the outcomes. Variations in surgeon experience and patient demographics at other institutions may also affect clinical results. Consequently, future multicenter studies with larger and more diverse populations are warranted to validate these findings. Second, outcomes related to wound satisfaction, such as incision size and subjective cosmetic assessment, were not systematically recorded. Furthermore, other patient-reported outcome measures, including pain intensity scores or passive range of motion, were not collected. However, the QuickDASH score provided a general evaluation of upper-extremity function, and the low scores observed in both groups suggest satisfactory functional recovery. It is also worth noting that the choice of implant typically determines the surgical approach and incision length in advance, and we believe that incision size variation was minimal. Previous studies have reported superior cosmetic results with ESIN compared to plate fixation [9]. Future prospective studies should include additional assessments to provide a more comprehensive evaluation of postoperative outcomes. Lastly, this study assessed outcomes only up to 1 year postoperatively. A longer follow-up will be necessary to more thoroughly evaluate long-term clinical outcomes.

## 5. Conclusions

The functional and radiographic outcomes of ESIN are comparable to those of plate fixation in adolescent diaphyseal clavicle fractures. It achieved fracture union approximately 3 weeks earlier and required a smaller surgical incision, while the postoperative complication rates were similar between the two groups. These findings suggest that ESIN is a safe and efficient method, comparable to plate fixation, for active adolescents.

## Figures and Tables

**Table 1:** Patient demographics and preoperative radiological parameters.

	ESIN (*n* = 18)	Plate Fixation (*n* = 17)	*p*-Value
	*n* (%) or Median (IQR)	*n* (%) or Median (IQR)	
Age (year)	14.8 (13.4, 15.2)	15.6 (14.5, 16.8)	0.307
Sex			
Male	16 (88.9)	16 (94.1)	0.581
Female	2 (11.1)	1 (5.9)	
BMI (kg/m^2^)	22.5 (18.9, 24.0)	22.4 (20.9, 24.1)	0.986
Fracture laterality			
Right	5 (27.8)	8 (47.1)	0.238
Left	13 (72.2)	9 (52.9)	
Injury mechanism ^1^			
Low	13 (72.2)	12 (70.6)	0.915
High	5 (27.8)	5 (29.4)	
Fracture type ^2^			
15-2A	7 (38.9)	6 (35.3)	0.826
15-2B/15-C	11 (61.1)	11 (64.7)	
Fracture shortening (mm)	21.0 (17.0, 23.0)	22.0 (12.0, 28.0)	0.944

ESIN, elastic stable intramedullary nail; IQR, interquartile range; BMI, body mass index. ^1^ High-energy defined as injuries resulting from road traffic accidents or falls from a height greater than 3 m. ^2^ Fracture type was classified by AO/OTA classification system; 15-A, as simple fracture, vs. 15-B, as wedge fracture/15-C, as segmental fracture.

**Table 2:** Postoperative outcomes followed by operative methods.

	ESIN (*n* = 18)	Plate Fixation (*n* = 17)	*p*-Value
	Median (IQR)	Median (IQR)	
QuickDASH score	0.0 (0.0, 4.5)	4.5 (0.0, 6.8)	0.339
Intraoperative time (min)	62.5 (55.0, 80.0)	65.0 (60.0, 80.0)	0.561
Time to complete fracture union ^1^ (week)	11.0 (8.0, 12.0)	14.0 (12.0, 16.0)	<0.001
Postoperative 1-year shortening (mm)	2.0 (0.0, 5.0)	0.0 (−3.0, 4.0)	0.411

ESIN, elastic stable intramedullary nail; IQR, interquartile range; QuickDASH, Quick Disabilities of the Arm, Shoulder, and Hand. ^1^ Complete cortical bridging on both anteroposterior and cephalic-tilt views between proximal and distal fracture fragment.

**Table 3:** Postoperative complications based on operative methods.

	ESIN (*n* = 18)	Plate Fixation (*n* = 17)	*p*-Value
	*n* (%)	*n* (%)	
Postoperative complication	5 (27.8)	4 (23.5)	0.774
Implant irritation	3 (16.7)	1 (5.9)	0.316
Chest wall numbness	0 (0.0)	1 (5.9)	0.297
Superficial wound infection	1 (5.6)	0 (0.0)	0.324
Refracture after implant removal	0 (0.0)	2 (11.8)	0.134
Implant bending	1 (5.6)	0 (0.0)	0.324

ESIN, elastic stable intramedullary nail.

## Data Availability

The dataset used and/or analyzed during the current study is available from the corresponding author upon reasonable request.

## Abbreviations

The following abbreviations are used in this manuscript:

ESIN	Elastic Stable Intramedullary Nailing
IRB	Institutional Review Board
BMI	Body Mass Index
AP	Anteroposterior
ICC	Intraclass Correlation Coefficient
QuickDASH	Quick Disabilities of the Arm, Shoulder, and Hand
IQR	Interquartile Range
LCP	Locking Compression Plate
SPSS	Statistical Package for the Social Sciences

## References

[1] Postacchini F., Gumina S., De Santis P., Albo F. (2002). Epidemiology of clavicle fractures. J. Shoulder Elb. Surg..

[2] Fanter N.J., Kenny R.M., Baker C.L., Baker C.L. (2015). Surgical treatment of clavicle fractures in the adolescent athlete. Sports Health.

[3] Ellis H.B., Li Y., Bae D.S., Kalish L.A., Wilson P.L., Pennock A.T., Nepple J.J., Willimon S.C., Spence D.D., Pandya N.K. (2020). Descriptive Epidemiology of Adolescent Clavicle Fractures: Results From the FACTS (Function after Adolescent Clavicle Trauma and Surgery) Prospective, Multicenter Cohort Study. Orthop. J. Sports Med..

[4] Hamid M.B.A., Younis Z., Mannan M., Prabhu R.M., Shrivastava N., Tauseef A., Nagaiah M.A., Raza A., Kashani A. (2025). Adolescent Clavicle Fractures: A Management Dilemma?. Cureus.

[5] Cole M.W., Collins L.K., Familia M.M., Skalak T.J., Lee O.C., Sherman W.F. (2023). Trends in the Treatment of Adolescent Clavicle Fractures: Are We Listening to the Evidence?. J. Am. Acad. Orthop. Surg. Glob. Res. Rev..

[6] Mitchell B.C., Ellis H., Wilson P., Pennock A.T. (2024). An Evidence-Based Approach to Managing Adolescent (Ages 10 to 19 Years) Diaphyseal Clavicle Fractures. J. Am. Acad. Orthop. Surg..

[7] Xu J., Xu L., Xu W., Gu Y., Xu J. (2014). Operative versus nonoperative treatment in the management of midshaft clavicular fractures: A meta-analysis of randomized controlled trials. J. Shoulder Elb. Surg..

[8] Canadian Orthopaedic Trauma Society (2007). Nonoperative treatment compared with plate fixation of displaced midshaft clavicular fractures. A multicenter, randomized clinical trial. J. Bone Jt. Surg. Am..

[9] Hong P., Liu R., Rai S., Ze R., Tang X., Li J. (2022). Plating versus elastic stable intramedullary nailing for displaced pediatric midshaft clavicular fractures. J. Orthop. Traumatol..

[10] Haider A.H., Chang D.C., Haut E.R., Cornwell E.E., Efron D.T. (2009). Mechanism of injury predicts patient mortality and impairment after blunt trauma. J. Surg. Res..

[11] Lima G.V., La Banca V., Murachovsky J., Nascimento L.G.P., Almeida L.H.O., Ikemoto R.Y. (2022). Assessment of the measurement methods in midshaft clavicle fracture. BMC Musculoskelet. Disord..

[12] Gabel C.P., Yelland M., Melloh M., Burkett B. (2009). A modified QuickDASH-9 provides a valid outcome instrument for upper limb function. BMC Musculoskelet. Disord..

[13] Quatman-Yates C.C., Gupta R., Paterno M.V., Schmitt L.C., Quatman C.E., Ittenbach R.F. (2013). Internal Consistency and Validity of the QuickDASH Instrument for Upper Extremity Injuries in Older Children. J. Pediatr. Orthop..

[14] McGraw M.A., Mehlman C.T., Lindsell C.J., Kirby C.L. (2009). Postnatal growth of the clavicle: Birth to 18 years of age. J. Pediatr. Orthop..

[15] Kamaci S., Bess L., Glogovac G., Colosimo A.J. (2022). Plate osteosynthesis of midshaft clavicle fractures in adolescent contact sports athletes-adolescent clavicle fracture. J. Pediatr. Orthop. B.

[16] Wright M., Della Rocca G.J. (2023). American Academy of Orthopaedic Surgeons Clinical Practice Guideline Summary on the Treatment of Clavicle Fractures. J. Am. Acad. Orthop. Surg..

[17] Houwert R.M., Wijdicks F.J., Steins Bisschop C., Verleisdonk E.J., Kruyt M. (2012). Plate fixation versus intramedullary fixation for displaced mid-shaft clavicle fractures: A systematic review. Int. Orthop..

[18] Wang Y.C., Fu Y.C., Chou S.H., Liu P.C., Tien Y.C., Lu C.C. (2015). Titanium Elastic Nail versus plate fixation of displaced midshaft clavicle fractures: A retrospective comparison study. Kaohsiung J. Med. Sci..

[19] Pennock A.T., Bae D.S., Boutelle K., Busch M.T., Carroll A., Edmonds E.W., Ellis H.B., Hergott K., Kocher M.S., Li Y. (2022). Remodeling of Adolescent Displaced Clavicle Fractures: A Facts Study. Orthop. J. Sports Med..

[20] Schulz J., Moor M., Roocroft J., Bastrom T.P., Pennock A.T. (2013). Functional and radiographic outcomes of nonoperative treatment of displaced adolescent clavicle fractures. J. Bone Jt. Surg. Am..

[21] Smekal V., Irenberger A., Attal R.E., Oberladstaetter J., Krappinger D., Kralinger F. (2011). Elastic stable intramedullary nailing is best for mid-shaft clavicular fractures without comminution: Results in 60 patients. Injury.

[22] Fuglesang H.F.S., Flugsrud G.B., Randsborg P.H., Oord P., Benth J., Utvåg S.E. (2017). Plate fixation versus intramedullary nailing of completely displaced midshaft fractures of the clavicle: A prospective randomised controlled trial. Bone Jt. J..

[23] Altay M.A., Erturk C., Cece H., Isikan U.E. (2011). Mini-open versus closed reduction in titanium elastic nailing of paediatric femoral shaft fractures: A comparative study. Acta Orthop. Belg..

[24] McKee R.C., Whelan D.B., Schemitsch E.H., McKee M.D. (2012). Operative versus nonoperative care of displaced midshaft clavicular fractures: A meta-analysis of randomized clinical trials. J. Bone Jt. Surg. Am..

[25] Eichinger J.K., Balog T.P., Grassbaugh J.A. (2016). Intramedullary Fixation of Clavicle Fractures: Anatomy, Indications, Advantages, and Disadvantages. J. Am. Acad. Orthop. Surg..

[26] Andrade-Silva F.B., Kojima K.E., Joeris A., Santos Silva J., Mattar R. (2015). Single, superiorly placed reconstruction plate compared with flexible intramedullary nailing for midshaft clavicular fractures: A prospective, randomized controlled trial. J. Bone Jt. Surg. Am..

[27] Kadakia A.P., Rambani R., Qamar F., McCoy S., Koch L., Venkateswaran B. (2012). Titanium elastic stable intramedullary nailing of displaced midshaft clavicle fractures: A review of 38 cases. Int. J. Shoulder Surg..

[28] Smekal V., Irenberger A., Struve P., Wambacher M., Krappinger D., Kralinger F.S. (2009). Elastic stable intramedullary nailing versus nonoperative treatment of displaced midshaft clavicular fractures-a randomized, controlled, clinical trial. J. Orthop. Trauma.

[29] van der Meijden O.A., Houwert R.M., Hulsmans M., Wijdicks F.-J.G., Dijkgraaf M.G.W., Meylaerts S.A.G., Hammacher E.R., Verhofstad M.H.J., Verleisdonk E.J.M.M. (2015). Operative Treatment of Dislocated Midshaft Clavicular Fractures: Plate or Intramedullary Nail Fixation?: A Randomized Controlled Trial. J. Bone Jt. Surg..

[30] Wang K., Dowrick A., Choi J., Rahim R., Edwards E. (2010). Post-operative numbness and patient satisfaction following plate fixation of clavicular fractures. Injury.

[31] Beirer M., Postl L., Crönlein M., Siebenlist S., Huber-Wagner S., Braun K.F., Biberthaler P., Kirchhoff C. (2015). Does a minimal invasive approach reduce anterior chest wall numbness and postoperative pain in plate fixation of clavicle fractures?. BMC Musculoskelet. Disord..

[32] Li Y., Helvie P., Farley F.A., Abbott M.D., Caird M.S. (2018). Complications After Plate Fixation of Displaced Pediatric Midshaft Clavicle Fractures. J. Pediatr. Orthop..

[33] Smith S.D., Wijdicks C.A., Jansson K.S., Boykin R.E., Martetschlaeger F., de Meijer P.P., Millett P.J., Hackett T.R. (2014). Stability of mid-shaft clavicle fractures after plate fixation versus intramedullary repair and after hardware removal. Knee Surg. Sports Traumatol. Arthrosc..

[34] James J., Ogden A., Mukherjee D., Jaeblon T. (2015). Residual Hole Orientation After Plate Removal: Effect on the Clavicle. Orthopedics.

[35] Hulsmans M.H., van Heijl M., Houwert R.M., Burger B.J., Verleisdonk E.J.M., Veeger D.J., van der Meijden O.A. (2018). Surgical fixation of midshaft clavicle fractures: A systematic review of biomechanical studies. Injury.

